# Mothers’ experience of disrespect and abuse during maternity care in northern Ethiopia

**DOI:** 10.1080/16549716.2018.1465215

**Published:** 2018-06-04

**Authors:** Mengistu Welday Gebremichael, Alemayehu Worku, Araya Abrha Medhanyie, Yemane Berhane

**Affiliations:** a College of Health Sciences, Mekelle University, Mekelle, Ethiopia; b School of Public Health, Addis Ababa University, Addis Ababa, Ethiopia; c Addis Continental Institute of Public Health, Addis Ababa, Ethiopia

**Keywords:** Gender and Health Inequality, Antenatal care, companionship, labour and delivery, respectful care, Tigray

## Abstract

**Background**: The provision of respectful and satisfactory maternity care is essential for promoting timely care-seeking behaviour, and ultimately ensuring the health and well-being of mothers and their babies. Disrespectful and abusive care has been recognized as one of the barriers to seeking timely maternity health services. However, the issue has not been adequately researched in community settings in low- and middle-income countries using validated measurement tools.

**Objective**: This study was conducted to assess the extent of, and factors associated with, disrespectful and abusive maternity care reported by women who utilized facility-based delivery services in northern Ethiopia.

**Methods**: We conducted a community-based cross-sectional study in Tigray, northern Ethiopia. Women who gave birth in the preceding year and visited health institutions for these deliveries were selected using a multistage cluster sampling procedure. Data were collected using a pretested questionnaire. Six domains of disrespect and abuse (D and A) were included in the questionnaire. Socio-demographic and obstetric related factors associated with D and A were tested using a negative binomial regression model.

**Results**: Of the 1125 women in the sample, 248 (22%; 95% CI: 19.8%, 24.4%) reported at least one incident of D and A during delivery at a public health facility in northern Ethiopia. Higher incidents of D and A were reported by women who were older than 19 years at the time of delivery (aIRR = 2.649 (95% CI: 1.455, 4.825) compared to younger women. Incidents of D and A were reported more by women residing in urban areas, by women educated to the ninth grade and above, by women who experienced longer labour duration, and also by women who were not permitted to have support persons attend labour and delivery.

**Conclusions**: A fifth of the women reported D and A while receiving care during labour and delivery. Policies and practices aimed at ensuring universal coverage for institutional deliveries need to promote respectful maternity care for women in all facilities.

## Background

The provision of respectful maternity services is a key strategy for preventing maternal suffering and deaths in sub-Saharan Africa [1,2]. Strategies such as locating birthing facilities close to communities and improving transportation are not sufficient inducements to encourage pregnant women to deliver in health facilities [3]. Individual and group perceptions and experiences in relation to the quality of care can influence the utilization of maternity services [4,5]. Improving the quality of maternity services and the manner in which they are delivered is critical for increasing service utilization [6,7].

**Figure 1.**
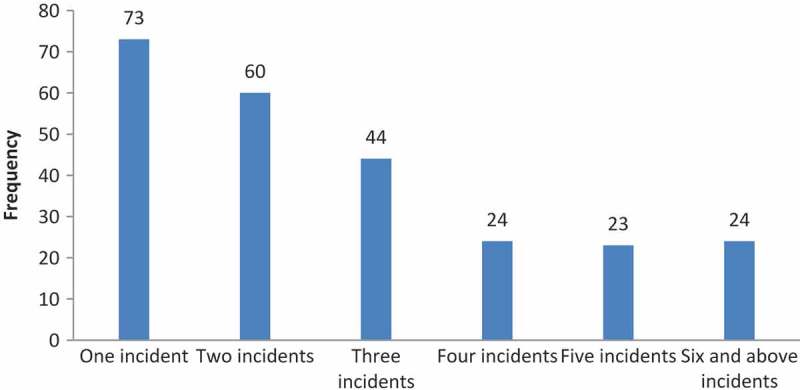
Incidents of D and A during labour and delivery in Tigray, Ethiopia, 2015.

The concept of ‘respectful maternity care’ is very difficult to measure because it is mainly dependent on women’s perceptions. As a result, this topic has been either ignored or under-reported [8]. Disrespectful and abusive care includes impoliteness of care providers, inappropriate reprimands, shouting at the client, lack of empathy, refusal to assist, threatening clients for their non-compliance, and denying clients opportunities to choose or give an opinion on the care they are receiving [9–11]. In recent years, the issue has received greater attention due to improved methods to operationally define and measure the phenomena [12–14]. However, community-based information on disrespectful and abusive care in sub-Saharan Africa is still lacking. Most previous studies were conducted in health institutions, where social desirability bias and fear of retaliatory action can potentially underestimate the extent of the problem. We assessed the quality of maternity care from users’ perspectives in order to bring women’s voices into the maternal health quality improvement agenda. This community-based study was conducted to assess the extent of, and factors associated with, disrespectful and abusive maternity care reported by women who utilized facility-based delivery services in northern Ethiopia.

## Methods

### Study setting

Tigray regional state in Ethiopia has 52 districts/woredas (34 rural and 18 urban) and 792 kebeles which are the lowest administrative units (722 rural and 70 urban kebeles) [15]. Within the region, there are 668 health posts, 218 health centres and 28 hospitals (11 primary, 16 general and 1 specialized). The general health service coverage is estimated to be 96%. In 2014, the regional health bureau report indicated 100% coverage for antenatal care, 55.3% for deliveries attended by skilled attendants, and 69.6% for postnatal care [15]. Maternity services in Ethiopia are provided free of charge in all government-owned health facilities. In this study area in northern Ethiopia, private health facilities rarely provide delivery services. Incidents reported here refer only to delivery facilities in government-run health facilities.

### Study participants

The study utilized a community based cross-sectional design. Women who gave birth in the preceding 12 months and visited public health facilities for birthing were the study subjects. This study period was chosen in order to achieve an adequate sample size and minimize recall bias. Women were selected for the study using a multistage sampling procedure. In the first stage, six districts were selected by simple random sampling. In the second stage, nine kebeles (three from urban and six from rural districts) were selected at random proportionate to the population size. The calculated sample size was proportionally allocated (736 to rural and 390 to urban kebeles). Rural kebeles with poor or no access to health services were excluded from the study sample. In the third stage, after selecting a random starting point, households with eligible women were sequentially enrolled until the allocated sample size for each selected kebele was achieved.

### Data collection tool and data collection

Data were collected, using an interviewer-administered questionnaire, over the period August to September 2015. The questionnaire was designed to collect information on socio-demographic characteristics, obstetric related conditions, and women’s experiences and perceptions of health services during labour and delivery. Based on Bowser and Hill’s report, this study measured six domains of disrespectful and abusive care which include: physical abuse, non-consensual care, non-confidential care, non-dignified care, discrimination based on specific patient attributes, and abandonment of care [8]. A seventh domain which captures information on detention was not included. Detainment in a health facility can occur because of failure to pay healthcare fees, and this study includes only maternity services provided without payment in government health facilities. The study instrument was first validated for content by experts, and then pretested on 50 women residing in similar settings outside the study area to check for ease of understanding and the time needed to complete the interview. The instrument was translated from English to the local language, Tigrigna, and then back-translated to English to ensure consistency in meaning.

Health extension workers, who are formal community-level primary healthcare providers, implemented the questionnaire in each village. This facilitated a high response rate because they were able to establish rapport with the mothers. Once a household with eligible women was identified there were up to three repeat visits to enroll the women in the study.

During data collection, the completeness of the questionnaires was checked on a daily basis by the field supervisors and the principal investigator. Data were entered using Epidata version 3.1 statistical software and then transferred to SPSS version 20 for analysis.

### Covariates

Information on residence, occupation, household head and permission for support persons to attend labour and delivery was captured and used to derive categorical variables. Maternal age was categorized into 3, as less than 20 years, 20–34, and 35 and above. Maternal educational status was categorized into 4, as illiterate, primary (1–4 grade), junior secondary (5–8 grade) and secondary and above. Total number of births to the mother were categorized into 3, as less than 3 births, 3–5 births, and 5 or more. Maternal age at marriage was categorized into 2, as 20 or less years, and more than 20 years. Duration of labour at the facility was categorized into 3, as less than 1 hour, 1–12 hours, 13–23 hours, and 24 or more hours.

The outcome of interest was count of occurrences of disrespectful and abusive practices/events, taking into account responses to all items in the questionnaire. An indicator variable was derived whereby respondents were classified as having experienced D and A if they reported at least one incident; women who scored zero were classified as not having experienced D and A.

### Analysis

Given that the outcome was a count, the appropriate model would have been a Poisson regression. However, the assumptions for this model were violated in terms of dispersion; the mean was 0.54, and the variance was 1.73 indicating the presence of over-dispersion. Negative Binomial Regression was chosen as the preferred model. The measure of association for the Negative Binomial Model is presented as the Incidence Rate Ratio and 95% confidence interval. Statistical significance was set at alpha 0.05. Variables were included in the multivariable Negative Binomial Model if their p-value was < 0.25 in bivariate analysis.

Data were missing at random for the economic variable, which referred to household income. However, this variable was not included in the analysis because the amount of missing data was > 10%. Missing data for all other variables was < 10%.

### Ethical consideration

Ethical clearance was obtained from Mekelle University Institutional Review Board (IRB). A written informed consent was obtained from each study participant; participants unable to sign were asked to give a thumbprint. Interviews were conducted in privacy and completed questionnaires were kept confidential and stored in a safe place.

## Results

Consistent with the sample size calculation, 1125 women were invited to participate in this study. All those invited agreed to participate. The majority of the women (902, 80.2%) were in the 20–34 years age category. The mean and median age of the women were 26.8 and 26.0 years, respectively. The majority of women were housewives (827, 73.5%) and rural residents (738, 65.6%). About one-third (415, 36.9%) of the women were illiterate (Table 1).

**Table 1.** Socio-demographic characteristics of study subjects, Tigray, Ethiopia, 2015.

Variables		Frequency	Percent
Age	less than 20 years	76	6.7
	20–34 years	902	80.2
	35 years and above	147	13.1
Ethnicity	Tigray	1115	99.1
	Erop	1	0.1
	Amhara	6	0.5
	Afar	3	0.3
Religion	Orthodox	995	88.4
	Muslim	125	11.1
	Catholic	4	0.4
	Protestant	1	0.1
Marital status	Married	1069	95
	Unmarried	17	1.5
	Divorced	35	3.1
	Widowed	4	0.4
Age at marriage	Less than 20 years	775	69.9
	20–34 years	333	30.1
Occupation	Housewife	827	73.5
	Farmer	105	9.3
	Self-employed	110	9.8
	Employee	62	5.5
	Other	21	1.9
Education	Illiterate	415	36.9
	Grade 1–4 (First cycle)	125	11.1
	Grade 5–8 (Second cycle)	248	22.0
	Grade 9–10 (Third cycle)	229	20.4
	Grade 11–12 and above (PCU and above)	108	9.6
Husband’s education	Illiterate	320	30.3
	Grade 1–4 (First cycle)	108	10.2
	Grade 5–8 (Second cycle)	205	19.4
	Grade 9–10 (Third cycle)	211	20.0
	Grade 11–12 and above (PCU and above)	213	20.1
Husbands’ occupation	Farmer	395	36.9
	Self-employed	326	30.5
	Employee	212	19.8
	Others	137	12.8
Total births	Less than 3	621	55.2
	1–3	402	35.7
	Greater than 5	102	9.1
Household size	2–5	801	71.2
	6 and above	324	28.8
Residence	Urban	387	34.4
	Rural	738	65.6
Head of household	Husband	1060	94.2
	Wife	65	5.8

Almost all study participants (1114, 99.1%) reported that they had visited a health institution for antenatal care services in the previous year, and 1031 (91.6%) reported that their last place of delivery was at a health facility. Out of 1125 women, 248 or 22% reported having experienced at least one type of D and A while receiving maternity services in health facilities. About 53% of participants reported that they were shouted at, 44.8% reported having been insulted or scolded and 33.9% reported being ignored when they requested services (Table 2). The majority of women reported two or more incidents (Figure 1).

**Table 2.** Women’s experience of D and A during labour and delivery services in Tigray, Ethiopia, 2015.

Variables		Frequency	Percent
**D and A during delivery services**			
Shouted at	Yes	132	12.5
	No	922	87.5
Scolded/insulted	Yes	111	10.5
	No	943	89.5
Discouraging/became negative to me	Yes	56	5.3
	No	998	94.7
Request for assistance/help ignored	Yes	84	8.0
	No	969	92.0
Not told information before/during a procedure	Yes	81	7.7
	No	973	92.3
Providers discussed my private health information in public	Yes	9	0.9
	No	1045	99.1
Shared my health information with others	Yes	8	0.8
	No	1046	99.2
Was made to lay on unhygienic bed/couch	Yes	40	3.8
	No	1014	96.2
Ashamed for being exposed naked to others	Yes	17	1.6
	No	1037	98.4
Movement restricted for a long time	Yes	40	3.8
	No	1014	96.2
Procedure/s done without being adequately informed	Yes	67	6.4
	No	987	93.6
Left alone unattended	Yes	63	6.0
	No	991	94.0
Hit/slapped/pushed by provider	Yes	8	0.8
	No	1046	99.2
Anything that you do not want to mention	Yes	2	0.2
	No	1052	99.8

In bivariable analyses, the variables religion, ethnicity, marital status, husband’s education, husband’s occupation, and levels of health facilities were dropped because in each case the p-value was > 0.25. Covariates retained for the multivariable analysis were residence, maternal age, maternal education, maternal occupation, total births, household head sex, number of individuals in household, age at marriage, number of  antenatal care (ANC) visits for the last pregnancy, duration of labour at facility and permission for support persons to attend labour and delivery.

In the multivariable analysis, D and A during delivery services was reported more among women residing in urban compared with rural areas (aIRR = 1.326; 95% CI: 1.018, 1.727) and women educated to grade 9 or above (aIRR = 1.487; 95% CI: 1.034, 2.139). Similarly, women in the age groups 20–34 (aIRR = 2.674; 95% CI: 1.468, 4.871), and 35 or above (aIRR = 2.892; 95% CI: 1.351, 6.189), compared to those below the age of 20 years, reported more incidents of D and A. Women who were heads of households reported more incidents of D and A (aIRR = 2.024; 95% CI: 1.201, 3.414) compared with women living in a household headed by a male. Women who had 3–5 births experienced fewer incidents of D and A (aIRR = 0.560; 95% CI: 0.318, 0.985) than women with more than 5 births (Table 3).

**Table 3.** Negative Binomial Regression estimates of incidence rate ratios of disrespectful and abusive care during labour and delivery with 95% confidence intervals (CIs) in Tigray, Ethiopia, 2015.

	Incidence Rate Ratios (Crude)	95% CIs		Incidence Rate Ratios (Adjusted)	95% CIs	
		Lower	Upper		Lower	Upper
**Residence** (Reference = Rural)	1			1		
Urban	1.626*	1.339	1.976	1.326*	1.018	1.727
**Age** (Reference = Less than 20 years)	1			1		
20–34	1.988*	1.251	3.161	2.674*	1.468	4.871
≥ 35	1.344	.785	2.302	2.892*	1.351	6.189
**Education** (Reference = Illiterate)	1			1		
Grade 1st–4th	1.270	.904	1.785	1.489	.980	2.263
Grade 5th–8th	1.280	.977	1.678	1.291	.909	1.836
9th grade and above	1.931*	1.527	2.442	1.487*	1.034	2.139
**Occupation** (Reference = Housewife)	1			1		
Farmer	.627*	.427	.920	.554	.189	1.626
Self-employed	1.120	.816	1.537	1.213	.787	1.869
Employee	1.824*	1.269	2.622	.870	.608	1.245
Other	.884	.423	1.849	.585	.332	1.032
**Total births** (Reference = > 5)	1			1		
<3	1.314	.928	1.861	.864	.456	1.638
3–5	0.797	0.550	1.156	0.560*	0.318	0.985
**Household head** (Reference = Husband)	1			1		
Wife	1.492*	1.031	2.158	2.024*	1.201	3.414
**Number of individuals in household** (Reference = > 5)	1			1		
2–5	1.589*	1.266	1.994	1.340	.905	1.984
**Age at marriage** (Reference = < 20 years)	1			1		
20–34 years	1.638*	1.341	2.001	1.009	.771	1.320
**Number of ANC visits for the last pregnancy** (Reference = ≥ 4 visits)	1			1		
One time	1.641	.866	3.110	.658	.206	2.097
Two times	1.042	.674	1.613	1.068	.583	1.953
Three times	1.087	.874	1.351	1.259	.960	1.650
**Duration of labour at facility**(Reference = < 1 hour)	1			1		
1 to 12	1.633*	1.115	2.393	1.262	.834	1.910
13 to 23	2.675*	1.658	4.315	1.755*	1.024	3.009
24 hours and more	4.091*	1.962	8.532	2.457*	1.075	5.619
**Permission for support persons to attend labour and delivery (**Reference = yes)	1			1		
No	2.400*	1.905	3.022	1.800*	1.383	2.344

Women who spent longer hours in labour in health facilities reported higher rates of D and A. Compared with women who spent less than 1 hour in labour, the adjusted incidence rate ratio for women who spent 13–23 hours in labour was 75% higher (aIRR = 1.755; 95% CI: 1.024, 3.009) and 2.5 times higher for women who spent 24 hours or more in labour (aIRR = 2.457; 95% CI: 1.075, 5.619). Women who were not permitted to have support persons/relatives in the delivery room also reported a significantly higher rate of D and A (aIRR = 1.800; 95% CI: 1.383, 2.344) during labour and delivery compared with those women who were allowed to have support persons (Table 3).

## Discussion

In this study, a fifth of women reported at least one incident of D and A while attending delivery services. Shouting, insults and ignoring women’s request for assistance were commonly reported types of disrespectful and abusive practices. Residence, women’s education, being the head of a household, the number of births, duration of labour at the health facility, and permission for support persons to attend labour and delivery were significantly associated with incidents of D and A.

In this study, the proportion of women (22%) who reported D and A during labour and delivery is similar to studies conducted in Kenya and Ethiopia [12,16], although lower than that reported in a study conducted in Nigeria (98%) [17]. The commonly reported incidents of D and A in this study were similar to those reported in many from sub-Saharan African countries [13]. These practices are totally unacceptable and unethical. Ignoring women’s requests for help could be very dangerous, leading to life-threatening complications and even death for either (or both) the mother and the baby [18,19].

Lack of information given to women before or during procedures, procedures conducted without their consent, and breach of privacy and confidentiality during childbirth are common practices associated with D and A in sub-Saharan Africa [14,16,20–22]. It is critical that healthcare providers offer women adequate explanation and obtain informed consent from women before administering treatment and performing procedures. These principles conform to fundamental human rights [23]. When women are well informed about the procedures they are required to undergo, their compliance with safety instructions is likely to be higher. They are also more likely to feel respected and come away with positive feelings about their childbirth experiences [9,22].

The proportion of women left unattended during labour (6%) was low in this study compared to that reported in other studies [12,14]. This could well be due to routine performance evaluations that were implemented in health services at the time of this study. Another reason was that the majority of the health institutions in the study also allowed women, but not men, to accompany women in the labour ward [21,23].

Educated women in urban study sites reported more incidents of D and A. Educated women are more knowledgeable about their rights and have higher expectations regarding their healthcare experiences than uneducated women [12,24]. Higher incidents of disrespectful and abusive care were reported from women who had longer labour durations. This may be related to longer observation times in uncomfortable circumstances, which may also cause severe distress and result in negative experiences. Health service users in developing countries generally have insufficient processes in place for appeals, and lengthy labour times provide more opportunities for negative experiences [25]. However, proper incident reporting can lead to improved policies and practices in health facilities and better informed health decision-making by women [26].

### Strengths of the study

This community-based study included participants from both rural and urban areas, interviewed outside health facilities. This gave women more freedom to express their feelings and report positive and negative experiences without fear, and eliminated social desirability bias [9]. The use of health extension workers who were known to the women and their communities was a factor in the study achieving a 100% response rate.

### Limitations of the study

We acknowledge recall and sampling bias as possible limitations. The recall period in this study was limited to one year in order to minimize recall errors; however, one year could still be considered too long to recall details of incidents that occurred during labour and delivery. There may also have been sampling bias due to the focus on a single encounter in the previous year. Only women who gave birth to live babies were included and therefore the study excluded stillbirths, neonatal and infant deaths. It is possible that those women may have had negative experiences of D and A, but this cannot be established from these data [9].

Women from rural study sites may have under-reported D and A due to their lack of awareness of their rights and having relatively lower expectations than urban women. On the other hand, women referred to a health facility as a result of life-threatening complications may feel gratitude and therefore downplay their reporting of incidents of D and A [9,12,27]. However, we cannot say whether this is true or not from these data.

We did not also include economic status in our analysis because of the large amount of missing data. This was possibly due to women having insufficient information about household income or being unable to report the value of their farm products in monetary terms. Information about facilities and whether there were procedures for reporting D and A was not collected. It is possible that we have underestimated the extent of D and A although we are unable to say whether this is the case.

## Conclusions

One in five women reported at least one incident of D and A during labour and delivery. It is important to strengthen accountability mechanisms and enable women to report any violations that occur during their use of health services. Further research is necessary to gain more information about this issue.

## Paper context

We conducted a community-based cross-sectional study in northern Ethiopia to investigate the extent of, and factors associated with, disrespectful and abusive maternity care reported by women. One-fifth of women reported disrespect and abuse while giving birth. Urban or educated women reported more incidents of disrespect and abuse. Women who had longer labour duration, and those who were not permitted to have support persons attend their labour and delivery, reported more incidents of disrespect and abuse.
